# The Siesta Habit is Associated with a Decreased Risk of Rupture of Intracranial Aneurysms

**DOI:** 10.3389/fneur.2017.00451

**Published:** 2017-09-01

**Authors:** Huibin Kang, Xin Feng, Baorui Zhang, Erkang Guo, Luyao Wang, Zenghui Qian, Peng Liu, Xiaolong Wen, Wenjuan Xu, Youxiang Li, Chuhan Jiang, Zhongxue Wu, Hongbing Zhang, Aihua Liu

**Affiliations:** ^1^Department of Neurosurgery, Beijing Luhe Hospital, Capital Medical University, Beijing, China; ^2^Department of Interventional Neuroradiology, Beijing Neurosurgical Institute, Capital Medical University, Beijing, China; ^3^Department of Interventional Neuroradiology, Beijing Tiantan Hospital, Capital Medical University, Beijing, China

**Keywords:** siesta, intracranial aneurysm, rupture, risk factor, assessment, confidence intervals

## Abstract

**Background:**

Previous studies have examined an association between the siesta habit and hypertension, as well as coronary heart disease. However, the relationship between a siesta and the risk of rupture of an intracranial aneurysm (IA) has not yet been established. We aimed to investigate the effects of a siesta on the risk of rupture of IAs.

**Methods:**

We prospectively enrolled consecutive patients diagnosed with IAs at our hospital between January 2016 and December 2016. Univariate and multivariate logistic regression analysis were performed to identify independent risk factors associated with IA rupture.

**Results:**

We studied 581 consecutive patients with 514 unruptured and 120 ruptured aneurysms. Univariate analysis demonstrated that hypertension, hyperlipidemia, diabetes mellitus, cigarette smoking, location, size, as well as shape and aspect ratio were associated with the risk of rupture of IAs. Multivariate analysis identified hypertension [odds ratio (OR) 1.68, 95% confidence interval (CI) 1.03–2.73], hyperlipidemia (OR 0.25, 95% CI 0.08–0.72), current cigarette smoking ≥20 cigarettes/day (d) (OR 3.48, 95% CI 1.63–7.47), siesta (siesta time <1 h, OR 0.49, 95% CI 0.24–0.98 and siesta time ≥1 h, OR 0.32, 95% CI 0.19–0.57), location of largest aneurysm on the anterior communicating and internal carotid-posterior communicating artery (PCOM) (anterior communicating artery OR 16.27, 95% CI 7.40–35.79 and PCOM OR 11.21, 95% CI 5.15–24.43), and size of aneurysm ≥7 mm (OR 2.19, 95% CI 1.21–3.97) as independent strong risk factors associated with risk of aneurysm rupture.

**Conclusion:**

In the present study, we found that a habitual siesta is a new predictive factor to assess the risk of rupture of an IA. We found the siesta habit may reduce the risk of aneurysm rupture. We also found that hypertension, hyperlipidemia, cigarette smoking, location, and size of aneurysm were associated with the risk of rupture of IAs.

## Introduction

While unruptured intracranial aneurysms (IAs) are found in 3–8% of the general population (1–3), only a few IAs are known to rupture (4). Approximately 2% of unruptured IAs present with subarachnoid hemorrhage (SAH) (4), which often results in death. Widespread use of neuroimaging techniques has led to detection of a greater number of incidental aneurysms (5). Although previous studies have identified demographic characteristics (*viz*., hypertension, smoking) and aneurysmal characteristics (*viz*., location, size) as independent risk factors associated with the risk of aneurysm rupture (6–12), predicting the risk of IA rupture remains controversial (13). Systemic hypertension has long been considered a significant risk factor linked to aneurysmal rupture (14, 15).

Particularly popular among older Chinese adults, a siesta is regarded a healthy habit. Appropriate napping can reduce systolic blood pressure, as well as decrease prevalence of hypertension in older adults (16). Previous studies have examined an association between the siesta habit and coronary heart disease and subclinical atherosclerosis (17, 18). However, to date, the relationship between the siesta habit and IA rupture has not been investigated. We aimed to identify the effects of the siesta habit on the risk of rupture of IA and other potential independent risk factors associated with IA rupture.

## Materials and Methods

### Ethics Statement

This study was carried out in accordance with the recommendations of the Institutional Review Board of Beijing Tiantan Hospital with written informed consent from all subjects. All subjects gave written informed consent in accordance with the Declaration of Helsinki.

### Patient Selection

An uninterrupted clinical database that had been developed at our hospital between January 2016 and December 2016 was reviewed retrospectively to identify all 581 patients with aneurysms (Table S1 in Supplementary Material).

Our exclusion criteria were: (1) fusiform, dissecting, traumatic, mycotic, or partially thrombosed aneurysms. (2) Intracranial hemorrhage of unknown cause, or aneurysms without clear and readable three-dimensional rotational angiography. (3) Aneurysms associated with cerebral arteriovenous fistulas, arteriovenous malformations, or moyamoya disease. (4) Patients who did not consent to participate in the study.

### Data Collection and Definitions

A semi-structured telephone survey conducted by trained interviewers was used to collect data pertaining to lifestyle including the siesta habit, duration of nocturnal sleep, smoking status, and alcohol consumption status. Other baseline data were obtained from the medical history recorded by treating physicians during interviews with patients and/or family members. Aneurysmal characteristics were obtained using three-dimensional rotational angiography and evaluated by two experienced neurosurgeons.

This study examined information regarding smoking, alcohol use, height, and body weight, the incidence of hypertension, hypercholesterolemia, diabetes mellitus, ischemic stroke, and heart disease, the time of siesta and nocturnal sleep. Smoking status was classified into four groups: (1) never a smoker, (2) former smoker (quit smoking before treatment), (3) current smoker (currently smoking ≥20 cigarettes/day), (4) current severe smoker (currently smoking ≥20 cigarettes/day). Alcohol consumption was defined as ≥18 U (*viz*., ≥150 g) per week (19). Height was measured in all subjects without shoes using a tape measure to the nearest 0.1 cm. Fasting body weight was measured in all subjects in light indoor clothing without shoes. The body mass index (kg m^−2^) was calculated as weight (kg) divided by height in square meters (m^2^) (16). A patient was considered hypertensive if he/she related history of using antihypertensive medications or history of unstable and untreated hypertension with systolic blood pressure ≥140 mmHg or diastolic blood pressure ≥90 mmHg. Patients treated with antihyperlipidemic agents or with a total cholesterol level ≥220 mg/dL were considered as having hypercholesterolemia. A patient was considered as having diabetes mellitus if he/she had history of using antidiabetic agents or insulin injections. A participant was considered as having heart disease if he/she had history of a myocardial infarction, angina pectoris, coronary arterial bypass grafting, or percutaneous transluminal coronary arterioplasty (20). Siesta was classified as those who did not take a siesta/nap (reference), short (<1 h) and long (≥1 h) per day over at least 3 days a week including weekdays and weekends (16). Nocturnal sleep was classified as short (<6 h, reference), medium (6–7 h) and long (≥7 h) per day. All participants underwent conventional digital subtraction angiography imaging to include the bilateral internal carotid arteries, bilateral external carotid arteries, and bilateral vertebral arteries for assessment of location, maximal size, and number of aneurysms.

### Definition of Parameters and Measurement

Aspect ratio (AR) was defined as the ratio of the maximum perpendicular height to the average neck diameter of the aneurysm. The average neck diameter was calculated as twice the distance from the neck centroid to the edge of the neck (21, 22). Size ratio (SR) was defined as the ratio of maximum aneurysm height to average diameter of parent artery (23).

### Statistical Analyses

The Statistical Package for the Social Sciences (SPSS, version 22.0, Chicago, IL, USA) was used to analyze the association between clinical and aneurysmal characteristics and rupture risk. A two-sided *P* values <0.05 was considered as statistically significant. Variables were reported as mean ± SD, and examined by student’s *t*-tests. Categorical variables were analyzed using Pearson’s χ^2^ tests. Pearson chi-square test or Fisher’s exact test were used to evaluate categorical variables. Variables that were not normally distributed were analyzed using the Mann–Whitney *U*-test. As predetermined, variables with a *P* value of <0.05 in the univariate logistic regression analysis were evaluated in the multivariate analysis. Multivariate logistic regression analysis (forward stepwise conditional) was performed to identify independent risk factors associated with IA rupture.

## Results

Our study included 581 consecutive patients with 634 IAs, including 120 ruptured and 514 unruptured IAs. These patients included 219 men and 362 women. The mean age was 53.4 ± 10.9 years. Baseline demographic characteristics of patients are shown in Table 1. IAs were divided into a ruptured and unruptured group based on findings obtained using computed tomography. Table 2 summarizes baseline data regarding aneurysmal characteristics and risk factors associated with the case and control groups.

**Table 1:** Patient characteristics.

Characteristic	Ruptured aneurysms (120 patients)	Unruptured aneurysms (461 patients)	Total patients (581)
Age, mean ± SD, years	54.0 ± 11.2	53.3 ± 117.6	53.4 ± 10.9
Female sex (%)	68 (56.7)	294 (63.8)	362 (62.3)
History of hypertension (%)	69 (57.5)	207 (44.9)	276 (47.5)
History of hyperlipidemia (%)	5 (4.2)	48 (10.4)	53 (9.1)
History of diabetes mellitus (%)	5 (4.2)	52 (11.3)	57 (9.8)
History of ischemic stroke (%)	11 (9.2)	37 (8.0)	48 (8.3)
History of heart disease (%)	7 (5.8)	19 (4.1)	26 (4.5)
**Cigarette smoking, *n* (%)**			0.001
Never	73 (60.8)	392 (85.0)	465 (80.0)
Past	4 (3.3)	18 (3.9)	22 (3.8)
Current <20 cigarettes/day	11 (9.2)	17 (3.7)	28 (4.8)
Current ≥20 cigarettes/day	32 (26.7)	34 (7.4)	66 (11.4)
Alcohol consumption, *n* (%)	39 (32.5)	77 (16.7)	116 (20.0)
**Body mass index (kg/m^2^)**			
Mean ± SD	25.0 ± 4.0	25.3 ± 3.9	25.3 ± 3.9
<24 (%)	53 (44.2)	181 (39.3)	234 (40.3)
24–26 (%)	26 (21.7)	107 (23.2)	133 (22.9)
≥26 (%)	41 (34.2)	173 (37.5)	214 (36.8)
**Siesta, ***h*** (%)**			
Never	50 (41.7)	110 (23.9)	160 (27.5)
<1 (%)	21 (17.5)	83 (18.0)	104 (17.9)
≥1 (%)	49 (40.8)	268 (58.1)	317 (68.8)
**Nocturnal sleep, ***h*** (%)**			
<6 (%)	39 (32.5)	181 (39.3)	220 (37.9)
6–7 (%)	41 (34.2)	113 (24.5)	154 (26.5)
≥7 (%)	40 (33.3)	167 (36.2)	207 (35.6)
Multiple unruptured aneurysms (%)	3 (2.5)	30 (6.5)	33 (5.7)

**Table 2:** Aneurysmal characteristics.

Characteristic	Ruptured aneurysms (120)	Unruptured aneurysms (514)	Total aneurysms (634)
**Location of largest aneurysm (%)**			
Internal carotid artery	11 (9.2)	245 (47.7)	256 (40.4)
Anterior cerebral artery	4 (3.3)	23 (4.5)	27 (4.3)
Middle cerebral artery	11 (9.2)	61 (11.9)	72 (11.4)
Anterior communicating artery	45 (37.5)	46 (8.9)	91 (14.4)
Internal carotid-posterior communicating artery	35 (29.2)	60 (11.7)	95 (15.0)
Vertebral artery-posterior inferior cerebellar artery and vertebrobasilar junction	14 (11.7)	79 (15.4)	93 (14.7)
**Diameter of largest aneurysm (mm)**			
<7	79 (65.8)	405 (78.8)	484 (76.3)
≥7	41 (34.2)	109 (21.2)	150 (23.7)
**Aneurysm shape (%)**			
Regular	31 (25.8)	213 (41.4)	244 (38.5)
Irregular	89 (74.2)	301 (58.6)	390 (61.5)
Size ratio	2.0 ± 1.6	2.0 ± 1.7	2.0 ± 1.6
Aspect ratio	1.5 ± 0.8	1.4 ± 0.9	17.0 ± 2.0
**Flow angles in degrees (%)**			
<90	57 (47.5)	201 (39.1)	258 (40.7)
≥90	63 (52.5)	313 (60.9)	376 (59.3)

### Univariate and Multivariate Analyses

Univariate analysis revealed that the presence of hypertension, hyperlipidemia, diabetes mellitus, current cigarette smoking, alcohol consumption, the siesta habit, location of the largest aneurysm, large-sized aneurysms (≥7 mm), aneurysm shape (irregular), and AR were significantly associated with IA rupture (*P* < 0.05, Table 3). Results of a multivariate logistic regression model using a backward stepwise method are shown in Table 2. Adjusted ORs and 95% CI for factors associated with IA rupture are shown in Table 3. Backward stepwise multivariate logistic regression analyses indicated that hypertension [odds ratio (OR) 1.68, 95% CI 1.03–2.73], hyperlipidemia (OR 0.25, 95% CI 0.08–0.72), current light smoker <20 cigarettes/day (OR 3.44, 95% CI 1.18–10.10), current severe smoker ≥20 cigarettes/day (OR 3.48, 95% CI 1.63–7.47), siesta ≥1 h (OR 0.49, 95% CI 0.24–0.98), siesta ≥1 h (OR 0.32, 95% CI 0.19–0.57), location of aneurysm on the anterior communicating artery (ACOM) (OR 16.27, 95% CI 7.40–35.79), and internal carotid-posterior communicating artery (PCOM) (OR 11.21, 95% CI 5.15–24.43) and diameter of largest aneurysm (≥7 mm) (OR 2.19, 95% CI 1.21–3.97) were significantly associated with risk of IA rupture (*P* < 0.05, Table 3).

**Table 3:** Univariate and multivariate regression analysis for rupture of intracranial aneurysms.

Characteristic	Univariate analysis hazard ratio (95% CI)	Multivariate analysis hazard ratio (95% CI)
Age	1.00 (0.98–1.02)	–
Female sex	0.74 (0.50–1.12)	–
History of hypertension	1.67 (1.11–2.50)	1.68 (1.03–2.73)
History of hyperlipidemia	0.37 (0.15–0.96)	0.25 (0.08–0.72)
History of diabetes mellitus	0.34 (0.13–0.88)	0.42 (0.17–1.06)
History of ischemic stroke	1.16 (0.57–2.34)	–
History of heart disease	1.44 (0.59–3.51)	–
**Cigarette smoking, *n***		
Never	Reference	Reference
Past	1.06 (0.35–3.16)	0.83 (0.22–3.24)
Current <20 cigarettes/day	3.21 (1.47–7.02)	3.44 (1.18–10.10)
Current ≥20 cigarettes/day	5.23 (3.07–8.92)	3.48 (1.63–7.47)
Alcohol consumption	2.40 (1.53–3.78)	1.49 (0.73–3.04)
**Body mass index (kg/m^2^)**		
Mean ± SD	0.98 (0.92–1.03)	–
<24	Reference	
24–26	0.84 (0.50–1.42)	–
≥26	0.87 (0.56–1.35)	–
**Siesta, *h***		
Never	Reference	Reference
<1	0.56 (0.31–0.99)	0.49 (0.24–0.98)
≥1	0.40 (0.26–0.63)	0.32 (0.19–0.57)
**Nocturnal sleep, *h***		
<6	Reference	
6–7	1.60 (0.97–2.63)	–
≥7	1.08 (0.67–1.76)	–
Multiple unruptured aneurysms	0.37 (0.11–1.23)	–
**Location of largest aneurysm**		
Internal carotid artery	Reference	Reference
Anterior cerebral artery	3.87 (1.14–13.14)	3.49 (0.95–12.89)
Middle cerebral artery	4.02 (1.67–9.70)	6.44 (2.70–15.37)
Anterior communicating artery	21.79 (10.49–45.24)	16.27 (7.40–35.79)
Internal carotid-posterior communicating artery	12.99 (6.24–27.07)	11.21 (5.15–24.43)
Vertebral artery-posterior inferior cerebellar artery and vertebrobasilar junction	3.95 (1.72–9.05)	3.07 (1.27–7.41)
Diameter of largest aneurysm (≥7 mm)	1.93 (1.25–2.97)	2.19 (1.21–3.97)
Aneurysm shape (irregular)	2.03 (1.30–3.17)	1.56 (0.93–2.60)
Size ratio	1.01 (0.90–1.14)	–
Aspect ratio	1.23 (1.00–1.52)	1.02 (0.75–1.37)
Flow angles (≥90°)	0.71 (0.48–1.06)	–

## Discussion

In the present study, we found that the siesta habit is a new protective factor to assess the risk of IA rupture—the siesta habit can reduce the risk of aneurysm rupture. We also found that hypertension, hyperlipidemia, cigarette smoking, as well as the location, and diameter of the largest aneurysm were associated with the risk of rupture of IAs.

Several research studies demonstrate that hypertension is associated with an increased risk of aneurysmal rupture (15, 24, 25). In 2014, Tada et al. (15) described an animal model of IAs to evaluate the role of systemic hypertension and the renin–angiotensin system in aneurysmal rupture. These authors found that normalization of blood pressure after formation of an aneurysm prevented aneurysmal rupture in mice and that inhibition of the local renin–angiotensin system could also prevent aneurysmal rupture independent of the reduction of blood pressure. Hypertension could produce multiple effects as follows: (1) the wall of a formed aneurysm may be weakened due to a direct increase in mechanical stresses, (2) systemic hypertension causes activation of the local renin–angiotensin system leading to vascular inflammation and remodeling and thereby contributes to aneurysmal rupture (26), (3) reportedly, certain polymorphisms in genes related to the renin–angiotensin system are associated with aneurysm rupture (15). Starke et al. (27) found that the tumor necrosis factor (TNF-α) plays a majoir role in the formation and rupture of aneurysms and that hypertension in conjunction with hemodynamic stress in rats *in vivo* increases the expression of TNF-α. Therefore, hypertension may directly or indirectly contribute to aneurysmal rupture. Additionally, our study showed that hypertension was an independent risk factor associated with a risk of IA rupture with an OR 1.68 and 95% CI 1.03–2.73.

Siesta or a noon nap (wu jiao in Chinese) is a common practice prevalent in China, Mediterranean countries, and in Latin America and is considered part of a healthy lifestyle in Chinese culture (28). Most old people in China take a habitual siesta for its health benefits (29). Cai et al. (16) found that a siesta was an independent risk factor associated with a lower prevalence of hypertension in older adults. A recent study revealed that a daily nap may accelerate cardiovascular recovery following mental stresses and additionally provide cardiovascular benefits and significantly lower mean arterial pressure were found during the recovery phase of the stress reactivity task among participants who received more than 45 min of daytime sleep (17). These studies demonstrate that it is reasonable to deduce that a siesta directly affects blood pressure and hypertension through the mechanism of cardiovascular recovery. A study found that adipocyte fatty acid-binding protein (aP2 or FABP4) is an adipokine known to be associated with an elevated blood pressure (30). Serum concentrations of adipocyte fatty acid-binding protein were lower in those taking a nap compared to subjects who could be classified as non-siesta subjects (16). These studies suggest that adipocyte fatty acid-binding protein probably has some role in the association between siesta and hypertension. However, to date, a relationship between the siesta habit and IA rupture has not been reported. In our study, we found that a siesta is a protective factor to help assess the risk of IA rupture and that the siesta habit can reduce the risk of aneurysm rupture. The OR for siesta <1 h was 0.49 (0.24–0.98), siesta ≥1 h was 0.32, 95% CI 0.19–0.57. A siesta may indirectly reduce the risk of aneurysmal rupture by lowering blood pressure. Further studies would be needed to understand the mechanisms by which a siesta affects the risk of aneurysm rupture.

Although the etiology is unclear, hypercholesterolemia may be associated with a reduced risk of SAH (9, 19). We found that hypercholesterolemia was a protective factor for IA rupture with an OR of 0.25, 95% CI 0.08–0.72. Cigarette smoking was an important risk factor associated with the risk of IA rupture. Many studies also report that smoking is an independent risk factor associated with aneurysmal rupture (20, 31–36). Long-term smoking affects vascular homeostasis and structural integrity of the vessel wall. Additionally, a deficiency of alpha 1-antitrypsin or one of the other protease inhibitors could cause degradation of the arterial wall due to an imbalance between proteolytic enzymes and their inhibitors, thereby predisposing the arterial wall to dissection or aneurysm formation (37–39). Our study showed that current smokers who smoked >20 cigarettes per day demonstrated a higher incidence of ruptured IA. Previous studies have reported that IAs located along the ACOM (9, 40) or PCOM (6, 9, 41) were significantly associated with the risk of rupture. In our study, ACOM (OR 16.27, 95% CI 7.40–35.79) and PCOM (OR 11.21, 95% CI 5.15–24.43) showed the highest incidence of rupture compared to those in other locations. Our study results matched those of previous studies, which demonstrate that aneurysm diameter ≥7 mm independently predicted subsequent aneurysm rupture (40, 42). The OR for aneurysmal diameter of the largest aneurysm (≥7 mm) was 2.19, 95% CI 1.21–3.97.

Factors that reportedly increase the risk of aneurysm rupture are the patient’s age, sex, multiple unruptured aneurysms, shape, SR, AR, and flow angles (10, 43–51), which may be independent risk factors for aneurysm rupture. However, in this study, these risk factors were non-significant.

### Limitations

The limitations of our study are: (1) our data did not include information regarding the quality of siesta and sleep such as initial insomnia and sleep fragmentation. It cannot be denied that the quality of a siesta and sleep may affect the association between siesta and aneurysm rupture, (2) ours was a single-center retrospective study with the possibility of a selection and referral bias. Our findings are entirely based on assessment of a Chinese population, (3) the effect of aneurysm rupture and subsequent hemorrhage on aneurysm geometry was not considered, although several studies suggest that rupture does not significantly alter aneurysm morphology (23, 52, 53), (4) previous studies suggested an annual risk of rupture of 0.34–1.3% (40, 54–56) among unruptured aneurysms, so there is inevitably unequal cohort sizes of ruptured and unruptured IAs, which might have influenced our results.

## Conclusion

Siesta is a new protective factor for assessment of the risk of IA rupture, and we could demonstrate that the siesta habit can reduce the risk of aneurysm rupture. We also found that hypertension, hyperlipidemia, cigarette smoking, location, and diameter of the largest aneurysm were associated with the risk of rupture of IAs.

## Author Contributions

Conceived and designed the experiments: AL, HZ. Performed the experiments: HK, XF. Analyzed the data: HK, XF, BZ. Contributed to reagents/materials/analysis tools: EG, ZQ, LW, PL, XW, WX, YL, and CJ. Wrote the paper: HK, XF. Revised the manuscript: ZW.

## Conflict of Interest Statement

The authors declare that the research was conducted in the absence of any commercial or financial relationships that could be construed as a potential conflict of interest.
