# Supplementary material for: The Siesta Habit is Associated with a Decreased Risk of Rupture of Intracranial Aneurysms
Source: Front Neurol. 2017 Sep 1;8:451. doi: 10.3389/fneur.2017.00451 (PMC5595157; doi:10.3389/fneur.2017.00451)
Supplement: Supplementary file 1 [file table_1.docx]

1. Ji W, Liu A, Lv X, et al. Risk score for neurological complications after endovascular treatment of unruptured intracranial aneurysms[J]. Stroke. 2016,47(4):971-978. doi:10.1161/STROKEAHA.115.012097

2. Ji, W., Liu, A., Yang, X., Li, Y., Jiang, C., & Wu, Z. (2016). Incidence and predictors of headache relief after endovascular treatment in patients with unruptured intracranial aneurysms. Interventional Neuroradiology Journal of Peritherapeutic Neuroradiology Surgical Procedures & Related Neurosciences.

Patients with dizzy (about 39.7%), intermittent headache (about 12.5%), symptoms of oppression (about 3.4%) …….

CT /MRI:no subarachnoid hemorrhage

Further examination:CTA/MRA

Unrupture aneurysms

Final examination: DSA

Patients with sudden severe headache

CT :subarachnoid hemorrhage

DSA

Rupture aneurysms
